# Terrestrial LiDAR-derived non-destructive woody biomass estimates for 10 hardwood species in Virginia

**DOI:** 10.1016/j.dib.2018.06.046

**Published:** 2018-06-22

**Authors:** Atticus E.L. Stovall, Kristina J. Anderson-Teixeira, Herman H. Shugart

**Affiliations:** a Department of Environmental Sciences, University of Virginia, Charlottesville, VA 22903, USA; b Conservation Ecology Center, Smithsonian Conservation Biology Institute; National Zoological Park, Front Royal, VA, USA; c Center for Tropical Forest Science-Forest Global Earth Observatory, Smithsonian Tropical Research Institute, Panama, Republic of Panama; d Conservation Ecology Center, USA

## Abstract

This article contains data related to the research article entitled “Assessing terrestrial laser scanning for developing non-destructive biomass allometry” (Stovall et al., 2018 [1]) and presents 258 terrestrial LiDAR-derived estimates of tree volume and biomass. The terrestrial LiDAR acquisitions were completed in the Center for Tropical Forest Science - Forest Global Earth Observatory (CTFS-ForestGEO) plot in Front Royal, Virginia, USA. The data includes tree diameter at breast height (DBH), total tree height, tree length (correcting for tree lean), average wood density, estimated wood volume, and dry weight or biomass for all trees. These data were used to develop aboveground biomass models [1] and the reader is referred to this study for additional information, interpretation, and reflection on applying this data.

**Specifications Table**

**Table:** 

Subject area	*Ecology*
More specific subject area	*Forestry*
Type of data	*Table*
How data was acquired	*Multi-position terrestrial laser scanning and 3D modeling with Computree and SimpleTree software.*
Data format	*Raw and processed*
Experimental factors	*Volume was estimated with 3D modeling using Computree and SimpleTree software. Volume estimates are approximately 5–15% accurate and nearly unbiased compared to destructive measurements. Average species-specific wood densities derived from*[2]*were applied to volume estimates for estimates of biomass. Independent validation of wood density shows error to be approximately 5%.*
Experimental features	*Data were collected focusing on the 10 dominant species at the site.*
Data source location	*Smithsonian Conservation Biology Institute near Front Royal, VA, USA (38°53׳36.6" N, 78°8׳43.4" W)*
Data accessibility	*Included with article*
Related research article	*Stovall, Atticus E. L., Texiera-Anderson, Kristina, Shugart, Herman H., Assessing terrestrial laser scanning for developing non-destructive biomass allometry. Forest Ecology and Management (2018) 427, 217-229.*

**Value of the data**

• Volumetric data are useful for studies of wood allocation in 10 dominant species on the East Coast of the United States.
• The data provide information useful for estimating forest carbon storage in hardwood forests in the United States.
• The data can be used as-is or combined with regionally similar measurements to create high sample size allometry including large diameter and height trees.

## 1 Data

**Fig. 1:**
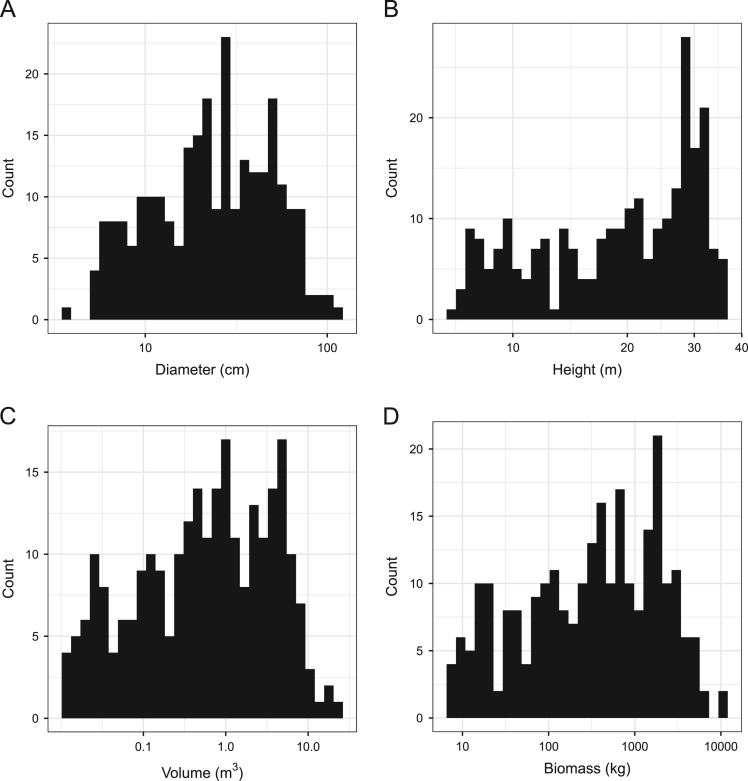
Log10-scaled summary distributions of tree-level [A] diameter, [B] height, [C] volume, and [D] biomass for use in allometric equation development.

We present data on terrestrial LiDAR-based tree-level estimates of diameter at breast height (DBH), height, length, volume, and biomass (Table 1; Fig. 1). Average species-specific wood density is presented for each individual measurement and was used to convert volume estimates into biomass estimates.

**Table 1:** Terrestrial LiDAR-derived diameter, length, height, and estimated volume and biomass values for 259 trees. Average wood density values [2] were used to convert volume estimates to biomass and are given for reference.

**Genus**	**Species**	**Wood Density (g/m**^**3**^**)**	**DBH (cm)**	**Length (m)**	**Height (m)**	**Volume (m**^**3**^**)**	**Biomass (kg)**
***Carya***	***Carya cordiformis***	0.62	7.71	8.38	7.96	0.0221	13.7
***Carya***	***Carya cordiformis***	0.62	11.50	10.87	10.80	0.1175	72.8
***Carya***	***Carya cordiformis***	0.62	12.39	7.29	7.11	0.0508	31.5
***Carya***	***Carya cordiformis***	0.62	12.87	14.03	13.87	0.1219	75.6
***Carya***	***Carya cordiformis***	0.62	17.80	13.53	13.49	0.1028	63.7
***Carya***	***Carya cordiformis***	0.62	18.19	14.99	14.76	0.5184	321.4
***Carya***	***Carya cordiformis***	0.62	22.87	25.50	25.20	0.7234	448.5
***Carya***	***Carya cordiformis***	0.62	26.89	14.45	14.37	0.7975	494.4
***Carya***	***Carya cordiformis***	0.62	29.23	26.85	26.68	1.3765	853.4
***Carya***	***Carya cordiformis***	0.62	36.19	29.70	29.51	1.9152	1187.4
***Carya***	***Carya cordiformis***	0.62	39.61	24.42	24.20	1.6606	1029.6
***Carya***	***Carya glabra***	0.62	3.85	7.97	7.90	0.0175	10.8
***Carya***	***Carya glabra***	0.62	5.03	7.98	7.94	0.0356	22.1
***Carya***	***Carya glabra***	0.62	5.10	7.77	7.66	0.0128	7.9
***Carya***	***Carya glabra***	0.62	5.87	8.45	8.16	0.0198	12.3
***Carya***	***Carya glabra***	0.62	6.00	8.35	8.30	0.0283	17.5
***Carya***	***Carya glabra***	0.62	6.00	8.52	8.34	0.0160	9.9
***Carya***	***Carya glabra***	0.62	6.05	9.31	9.22	0.0171	10.6
***Carya***	***Carya glabra***	0.62	6.12	9.45	9.25	0.0192	11.9
***Carya***	***Carya glabra***	0.62	6.57	10.01	9.91	0.0272	16.9
***Carya***	***Carya glabra***	0.62	6.68	7.69	7.63	0.0171	10.6
***Carya***	***Carya glabra***	0.62	7.03	7.81	7.69	0.0237	14.7
***Carya***	***Carya glabra***	0.62	7.34	8.63	7.61	0.0269	16.7
***Carya***	***Carya glabra***	0.62	7.35	7.12	7.08	0.0271	16.8
***Carya***	***Carya glabra***	0.62	8.11	9.68	9.45	0.0292	18.1
***Carya***	***Carya glabra***	0.62	8.30	9.38	9.32	0.0294	18.2
***Carya***	***Carya glabra***	0.62	8.32	7.78	7.72	0.0128	7.9
***Carya***	***Carya glabra***	0.62	8.40	11.22	11.09	0.0891	55.2
***Carya***	***Carya glabra***	0.62	8.47	9.58	9.14	0.0303	18.8
***Carya***	***Carya glabra***	0.62	9.30	9.69	9.46	0.0523	32.4
***Carya***	***Carya glabra***	0.62	9.32	8.03	7.87	0.0353	21.9
***Carya***	***Carya glabra***	0.62	9.93	11.59	11.53	0.0962	59.6
***Carya***	***Carya glabra***	0.62	10.95	9.02	8.11	0.1580	98.0
***Carya***	***Carya glabra***	0.62	11.24	12.05	12.01	0.1309	81.2
***Carya***	***Carya glabra***	0.62	12.15	8.95	8.73	0.0681	42.2
***Carya***	***Carya glabra***	0.62	12.53	13.83	13.61	0.1687	104.6
***Carya***	***Carya glabra***	0.62	12.70	12.14	11.85	0.1788	110.8
***Carya***	***Carya glabra***	0.62	12.91	12.79	12.75	0.1348	83.6
***Carya***	***Carya glabra***	0.62	13.28	15.21	15.18	0.1433	88.8
***Carya***	***Carya glabra***	0.62	16.12	11.90	11.39	0.1546	95.9
***Carya***	***Carya glabra***	0.62	16.35	13.40	13.38	0.1653	102.5
***Carya***	***Carya glabra***	0.62	16.47	11.64	11.21	0.1582	98.1
***Carya***	***Carya glabra***	0.62	16.50	19.32	19.24	0.2124	131.7
***Carya***	***Carya glabra***	0.62	17.90	17.58	17.45	0.3598	223.1
***Carya***	***Carya glabra***	0.62	18.21	20.92	20.43	0.3606	223.6
***Carya***	***Carya glabra***	0.62	18.73	16.15	16.02	0.2903	180.0
***Carya***	***Carya glabra***	0.62	20.00	19.53	19.44	0.4231	262.3
***Carya***	***Carya glabra***	0.62	20.70	17.89	17.81	0.3278	203.2
***Carya***	***Carya glabra***	0.62	20.97	21.43	20.78	0.4328	268.4
***Carya***	***Carya glabra***	0.62	21.60	22.37	22.36	0.4932	305.8
***Carya***	***Carya glabra***	0.62	22.05	16.47	16.31	0.6298	390.5
***Carya***	***Carya glabra***	0.62	22.05	19.04	18.75	0.5651	350.3
***Carya***	***Carya glabra***	0.62	22.15	21.15	21.06	0.5966	369.9
***Carya***	***Carya glabra***	0.62	23.00	24.29	24.27	0.8904	552.0
***Carya***	***Carya glabra***	0.62	23.52	18.40	18.14	0.4994	309.6
***Carya***	***Carya glabra***	0.62	23.68	16.37	15.77	0.3855	239.0
***Carya***	***Carya glabra***	0.62	24.85	18.34	18.26	0.6733	417.4
***Carya***	***Carya glabra***	0.62	25.10	23.79	23.47	0.9752	604.6
***Carya***	***Carya glabra***	0.62	25.28	23.66	23.45	0.8346	517.5
***Carya***	***Carya glabra***	0.62	26.35	20.96	20.78	0.7954	493.1
***Carya***	***Carya glabra***	0.62	26.84	22.19	21.86	0.7858	487.2
***Carya***	***Carya glabra***	0.62	27.02	24.77	24.75	1.0007	620.4
***Carya***	***Carya glabra***	0.62	29.40	25.31	25.15	0.9404	583.0
***Carya***	***Carya glabra***	0.62	30.78	23.19	22.77	1.1520	714.2
***Carya***	***Carya glabra***	0.62	32.99	26.56	26.08	1.3835	857.8
***Carya***	***Carya glabra***	0.62	34.91	20.37	20.33	1.1856	735.1
***Carya***	***Carya glabra***	0.62	35.46	26.74	26.34	1.1094	687.8
***Carya***	***Carya glabra***	0.62	35.89	30.10	29.87	2.0202	1252.5
***Carya***	***Carya glabra***	0.62	36.90	32.10	31.71	2.0937	1298.1
***Carya***	***Carya glabra***	0.62	38.00	28.67	28.55	2.3327	1446.3
***Carya***	***Carya glabra***	0.62	39.24	30.34	30.14	1.9271	1194.8
***Carya***	***Carya glabra***	0.62	42.10	27.02	26.95	2.7890	1729.1
***Carya***	***Carya glabra***	0.62	46.51	29.64	28.88	3.3860	2099.3
***Carya***	***Carya ovalis***	0.62	6.50	7.48	7.31	0.0141	8.8
***Carya***	***Carya ovalis***	0.62	9.51	12.65	12.39	0.0561	34.8
***Carya***	***Carya ovalis***	0.62	11.27	11.77	11.74	0.1032	64.0
***Carya***	***Carya ovalis***	0.62	14.35	17.30	17.17	0.1927	119.5
***Carya***	***Carya ovalis***	0.62	18.50	20.40	20.25	0.3452	214.0
***Carya***	***Carya ovalis***	0.62	18.72	17.91	17.72	0.4297	266.4
***Carya***	***Carya ovalis***	0.62	22.59	20.47	20.31	0.8479	525.7
***Carya***	***Carya ovalis***	0.62	22.71	22.16	21.91	0.5496	340.7
***Carya***	***Carya ovalis***	0.62	27.19	26.26	26.14	1.1507	713.4
***Carya***	***Carya ovalis***	0.62	27.42	22.25	22.12	0.8586	532.3
***Carya***	***Carya ovalis***	0.62	27.89	27.63	27.59	0.9710	602.0
***Carya***	***Carya ovalis***	0.62	28.89	21.27	21.02	0.6063	375.9
***Carya***	***Carya ovalis***	0.62	37.35	28.12	27.94	2.6495	1642.7
***Carya***	***Carya ovalis***	0.62	38.39	24.11	23.52	2.1089	1307.5
***Carya***	***Carya ovalis***	0.62	45.50	28.29	27.96	2.3852	1478.8
***Carya***	***Carya tomentosa***	0.62	5.21	7.67	7.36	0.0126	7.8
***Carya***	***Carya tomentosa***	0.62	5.48	9.97	9.95	0.0255	15.8
***Carya***	***Carya tomentosa***	0.62	5.72	13.38	13.26	0.0460	28.5
***Carya***	***Carya tomentosa***	0.62	6.09	8.01	7.87	0.0155	9.6
***Carya***	***Carya tomentosa***	0.62	6.68	9.20	8.71	0.0253	15.7
***Carya***	***Carya tomentosa***	0.62	6.80	9.82	9.79	0.0248	15.4
***Carya***	***Carya tomentosa***	0.62	6.80	7.59	7.58	0.0172	10.7
***Carya***	***Carya tomentosa***	0.62	7.31	10.97	10.48	0.0282	17.5
***Carya***	***Carya tomentosa***	0.62	7.66	8.37	8.21	0.0131	8.1
***Carya***	***Carya tomentosa***	0.62	7.91	9.08	8.89	0.0285	17.7
***Carya***	***Carya tomentosa***	0.62	9.60	11.77	11.60	0.0710	44.0
***Carya***	***Carya tomentosa***	0.62	10.05	10.76	10.50	0.0872	54.1
***Carya***	***Carya tomentosa***	0.62	10.40	13.52	13.27	0.0743	46.0
***Carya***	***Carya tomentosa***	0.62	10.45	12.19	12.09	0.0599	37.2
***Carya***	***Carya tomentosa***	0.62	10.61	9.18	9.03	0.0295	18.3
***Carya***	***Carya tomentosa***	0.62	10.77	12.35	11.85	0.0781	48.4
***Carya***	***Carya tomentosa***	0.62	12.05	15.72	15.54	0.1109	68.7
***Carya***	***Carya tomentosa***	0.62	12.58	13.70	13.45	0.1119	69.4
***Carya***	***Carya tomentosa***	0.62	12.79	12.99	12.21	0.1235	76.6
***Carya***	***Carya tomentosa***	0.62	16.90	10.58	10.43	0.1860	115.3
***Carya***	***Carya tomentosa***	0.62	18.40	28.85	28.82	0.6269	388.7
***Carya***	***Carya tomentosa***	0.62	19.36	19.68	18.69	0.3544	219.8
***Carya***	***Carya tomentosa***	0.62	20.04	19.84	19.81	0.4635	287.4
***Carya***	***Carya tomentosa***	0.62	20.16	16.07	16.01	0.2499	154.9
***Carya***	***Carya tomentosa***	0.62	20.20	19.86	19.51	0.4128	255.9
***Carya***	***Carya tomentosa***	0.62	21.23	20.10	20.01	0.4712	292.1
***Carya***	***Carya tomentosa***	0.62	22.61	17.69	17.53	0.5398	334.7
***Carya***	***Carya tomentosa***	0.62	23.40	18.02	17.97	0.6152	381.4
***Carya***	***Carya tomentosa***	0.62	24.02	23.81	23.63	0.5427	336.5
***Carya***	***Carya tomentosa***	0.62	26.24	24.06	23.60	0.9872	612.1
***Carya***	***Carya tomentosa***	0.62	30.39	28.94	28.38	1.8986	1177.1
***Carya***	***Carya tomentosa***	0.62	31.62	28.39	28.02	1.0139	628.6
***Carya***	***Carya tomentosa***	0.62	41.07	29.26	29.11	2.6463	1640.7
***Fagus***	***Fagus grandifolia***	0.56	6.26	8.31	8.12	0.0210	11.8
***Fagus***	***Fagus grandifolia***	0.56	6.75	11.33	11.16	0.1120	62.7
***Fagus***	***Fagus grandifolia***	0.56	7.15	9.53	9.37	0.0360	20.2
***Fagus***	***Fagus grandifolia***	0.56	8.09	8.73	8.70	0.0338	18.9
***Fagus***	***Fagus grandifolia***	0.56	9.33	8.89	8.84	0.0391	21.9
***Fagus***	***Fagus grandifolia***	0.56	9.97	11.27	11.06	0.0696	39.0
***Fagus***	***Fagus grandifolia***	0.56	12.62	9.88	9.52	0.0673	37.7
***Fagus***	***Fagus grandifolia***	0.56	15.13	15.46	15.40	0.2102	117.7
***Fagus***	***Fagus grandifolia***	0.56	16.65	17.57	17.56	0.2597	145.4
***Fagus***	***Fagus grandifolia***	0.56	16.99	19.03	18.92	0.2874	160.9
***Fagus***	***Fagus grandifolia***	0.56	19.42	18.21	18.15	0.4179	234.0
***Fagus***	***Fagus grandifolia***	0.56	20.04	20.06	20.02	0.3798	212.7
***Fagus***	***Fagus grandifolia***	0.56	20.95	18.77	18.46	0.6326	354.2
***Fagus***	***Fagus grandifolia***	0.56	22.20	18.65	18.58	0.7128	399.2
***Fagus***	***Fagus grandifolia***	0.56	25.60	14.38	14.35	0.4182	234.2
***Fagus***	***Fagus grandifolia***	0.56	26.95	20.74	20.68	0.9607	538.0
***Fagus***	***Fagus grandifolia***	0.56	28.18	21.70	21.67	1.1058	619.2
***Fagus***	***Fagus grandifolia***	0.56	30.63	27.46	27.19	1.2568	703.8
***Fagus***	***Fagus grandifolia***	0.56	36.50	28.20	27.86	2.8419	1591.4
***Fagus***	***Fagus grandifolia***	0.56	104.68	33.95	33.66	19.9617	11,178.6
***Fraxinus***	***Fraxinus americana***	0.55	7.75	10.52	9.85	0.0424	23.3
***Fraxinus***	***Fraxinus americana***	0.55	9.65	9.60	9.57	0.0382	21.0
***Fraxinus***	***Fraxinus americana***	0.55	11.25	9.88	9.68	0.0563	30.9
***Fraxinus***	***Fraxinus americana***	0.55	11.30	10.24	10.26	0.0881	48.5
***Fraxinus***	***Fraxinus americana***	0.55	11.95	11.73	11.70	0.0883	48.6
***Fraxinus***	***Fraxinus americana***	0.55	12.89	13.56	13.47	0.1638	90.1
***Fraxinus***	***Fraxinus americana***	0.55	13.58	11.80	11.58	0.0845	46.5
***Fraxinus***	***Fraxinus americana***	0.55	14.55	13.93	13.87	0.1211	66.6
***Fraxinus***	***Fraxinus americana***	0.55	15.60	12.30	12.16	0.1917	105.5
***Fraxinus***	***Fraxinus americana***	0.55	18.24	13.83	12.13	0.2706	148.9
***Fraxinus***	***Fraxinus americana***	0.55	26.49	22.55	22.01	0.7270	399.8
***Fraxinus***	***Fraxinus americana***	0.55	26.65	21.21	21.04	0.8340	458.7
***Fraxinus***	***Fraxinus americana***	0.55	45.36	31.77	31.66	3.2010	1760.5
***Fraxinus***	***Fraxinus americana***	0.55	45.98	26.38	25.20	2.7579	1516.9
***Fraxinus***	***Fraxinus americana***	0.55	52.31	31.83	31.68	4.9595	2727.7
***Fraxinus***	***Fraxinus americana***	0.55	54.98	28.59	28.33	4.7316	2602.4
***Liriodendron***	***Liriodendron tulipifera***	0.4	9.91	10.34	10.21	0.0928	37.1
***Liriodendron***	***Liriodendron tulipifera***	0.4	13.12	13.76	9.83	0.1365	54.6
***Liriodendron***	***Liriodendron tulipifera***	0.4	16.19	14.63	14.57	0.2860	114.4
***Liriodendron***	***Liriodendron tulipifera***	0.4	16.49	14.67	14.36	0.2452	98.1
***Liriodendron***	***Liriodendron tulipifera***	0.4	17.79	17.25	17.25	0.2826	113.0
***Liriodendron***	***Liriodendron tulipifera***	0.4	17.95	17.87	17.42	0.4249	169.9
***Liriodendron***	***Liriodendron tulipifera***	0.4	18.43	19.07	18.87	0.3040	121.6
***Liriodendron***	***Liriodendron tulipifera***	0.4	18.65	14.81	14.78	0.3165	126.6
***Liriodendron***	***Liriodendron tulipifera***	0.4	19.14	19.87	19.78	0.3695	147.8
***Liriodendron***	***Liriodendron tulipifera***	0.4	20.78	14.78	14.75	0.3411	136.5
***Liriodendron***	***Liriodendron tulipifera***	0.4	22.55	19.01	18.44	0.4812	192.5
***Liriodendron***	***Liriodendron tulipifera***	0.4	26.21	22.28	22.10	0.7653	306.1
***Liriodendron***	***Liriodendron tulipifera***	0.4	26.27	27.50	27.50	1.1147	445.9
***Liriodendron***	***Liriodendron tulipifera***	0.4	28.00	28.84	28.74	1.0793	431.7
***Liriodendron***	***Liriodendron tulipifera***	0.4	28.63	21.79	21.48	0.8903	356.1
***Liriodendron***	***Liriodendron tulipifera***	0.4	31.75	27.49	26.61	1.1447	457.9
***Liriodendron***	***Liriodendron tulipifera***	0.4	32.48	28.73	28.43	1.1913	476.5
***Liriodendron***	***Liriodendron tulipifera***	0.4	34.82	29.33	29.28	1.5505	620.2
***Liriodendron***	***Liriodendron tulipifera***	0.4	36.60	29.87	29.72	1.7776	711.0
***Liriodendron***	***Liriodendron tulipifera***	0.4	39.97	28.23	28.21	2.4107	964.3
***Liriodendron***	***Liriodendron tulipifera***	0.4	40.45	31.17	31.15	2.9096	1163.8
***Liriodendron***	***Liriodendron tulipifera***	0.4	44.18	27.98	27.95	2.4287	971.5
***Liriodendron***	***Liriodendron tulipifera***	0.4	46.40	31.71	31.64	3.1674	1267.0
***Liriodendron***	***Liriodendron tulipifera***	0.4	46.70	32.38	32.02	3.5239	1409.6
***Liriodendron***	***Liriodendron tulipifera***	0.4	49.23	31.93	31.67	4.3199	1728.0
***Liriodendron***	***Liriodendron tulipifera***	0.4	49.27	35.08	34.97	4.1313	1652.5
***Liriodendron***	***Liriodendron tulipifera***	0.4	49.42	35.52	35.53	3.2531	1301.2
***Liriodendron***	***Liriodendron tulipifera***	0.4	50.18	30.20	30.00	4.6933	1877.3
***Liriodendron***	***Liriodendron tulipifera***	0.4	51.34	32.78	32.58	4.4997	1799.9
***Liriodendron***	***Liriodendron tulipifera***	0.4	51.39	32.70	32.24	4.0959	1638.4
***Liriodendron***	***Liriodendron tulipifera***	0.4	51.55	32.00	31.97	3.8751	1550.0
***Liriodendron***	***Liriodendron tulipifera***	0.4	52.05	31.36	31.35	4.4293	1771.7
***Liriodendron***	***Liriodendron tulipifera***	0.4	53.68	31.59	31.50	4.6408	1856.3
***Liriodendron***	***Liriodendron tulipifera***	0.4	54.32	32.21	32.06	4.0605	1624.2
***Liriodendron***	***Liriodendron tulipifera***	0.4	54.62	31.48	31.42	5.4405	2176.2
***Liriodendron***	***Liriodendron tulipifera***	0.4	57.21	34.59	34.30	5.1119	2044.7
***Liriodendron***	***Liriodendron tulipifera***	0.4	59.34	33.65	33.53	6.1902	2476.1
***Liriodendron***	***Liriodendron tulipifera***	0.4	59.73	32.22	31.59	6.6975	2679.0
***Liriodendron***	***Liriodendron tulipifera***	0.4	60.23	37.39	36.72	5.1696	2067.8
***Liriodendron***	***Liriodendron tulipifera***	0.4	63.87	37.19	36.72	6.3242	2529.7
***Liriodendron***	***Liriodendron tulipifera***	0.4	65.25	36.70	36.67	6.8760	2750.4
***Liriodendron***	***Liriodendron tulipifera***	0.4	71.69	33.35	32.62	5.7211	2288.4
***Liriodendron***	***Liriodendron tulipifera***	0.4	71.72	33.14	33.09	9.8612	3944.5
***Liriodendron***	***Liriodendron tulipifera***	0.4	72.65	36.64	36.43	8.0814	3232.6
***Liriodendron***	***Liriodendron tulipifera***	0.4	74.00	32.96	32.91	7.0123	2804.9
***Liriodendron***	***Liriodendron tulipifera***	0.4	82.11	32.57	32.52	9.9237	3969.5
***Liriodendron***	***Liriodendron tulipifera***	0.4	103.61	34.52	33.88	16.7140	6685.6
***Liriodendron***	***Liriodendron tulipifera***	0.4	120.09	34.12	33.95	25.1986	10,079.4
***Quercus***	***Quercus alba***	0.6	20.75	15.72	15.44	0.3391	203.4
***Quercus***	***Quercus alba***	0.6	24.29	20.93	20.92	0.7266	435.9
***Quercus***	***Quercus alba***	0.6	27.55	25.64	24.78	1.1062	663.7
***Quercus***	***Quercus alba***	0.6	28.92	25.11	24.94	1.3091	785.4
***Quercus***	***Quercus alba***	0.6	30.24	23.52	23.25	1.0483	629.0
***Quercus***	***Quercus alba***	0.6	33.64	26.18	21.72	1.0952	657.1
***Quercus***	***Quercus alba***	0.6	37.90	27.52	27.48	1.3259	795.5
***Quercus***	***Quercus alba***	0.6	39.32	25.25	24.92	2.2275	1336.5
***Quercus***	***Quercus alba***	0.6	46.43	27.68	27.04	3.4182	2050.9
***Quercus***	***Quercus alba***	0.6	48.52	28.22	27.99	4.1909	2514.6
***Quercus***	***Quercus alba***	0.6	48.77	29.91	28.77	2.6920	1615.2
***Quercus***	***Quercus alba***	0.6	48.80	27.32	26.63	4.2608	2556.5
***Quercus***	***Quercus alba***	0.6	49.35	28.11	27.77	2.4510	1470.6
***Quercus***	***Quercus alba***	0.6	51.40	30.16	29.73	5.9124	3547.4
***Quercus***	***Quercus alba***	0.6	54.48	31.23	30.84	3.9714	2382.9
***Quercus***	***Quercus alba***	0.6	58.39	31.63	31.54	5.2404	3144.2
***Quercus***	***Quercus alba***	0.6	59.24	31.01	30.21	7.7732	4663.9
***Quercus***	***Quercus alba***	0.6	61.78	27.38	27.29	6.2910	3774.6
***Quercus***	***Quercus alba***	0.6	65.55	31.57	30.92	8.9610	5376.6
***Quercus***	***Quercus alba***	0.6	65.58	28.24	27.71	5.5006	3300.4
***Quercus***	***Quercus alba***	0.6	67.25	29.84	29.74	6.1302	3678.1
***Quercus***	***Quercus alba***	0.6	68.38	28.22	28.18	4.8958	2937.5
***Quercus***	***Quercus alba***	0.6	69.00	24.61	24.36	4.6297	2777.8
***Quercus***	***Quercus alba***	0.6	72.57	29.56	29.43	8.2607	4956.4
***Quercus***	***Quercus alba***	0.6	76.25	32.12	31.57	7.7188	4631.3
***Quercus***	***Quercus prinus***	0.57	11.09	8.74	8.45	0.0529	30.2
***Quercus***	***Quercus prinus***	0.57	20.52	20.12	19.27	0.3394	193.5
***Quercus***	***Quercus prinus***	0.57	27.30	24.98	23.81	1.1316	645.0
***Quercus***	***Quercus prinus***	0.57	27.60	22.56	22.02	0.7023	400.3
***Quercus***	***Quercus prinus***	0.57	34.49	28.69	28.40	1.3449	766.6
***Quercus***	***Quercus prinus***	0.57	34.50	23.67	22.27	1.4853	846.6
***Quercus***	***Quercus prinus***	0.57	34.89	25.96	25.87	1.6700	951.9
***Quercus***	***Quercus prinus***	0.57	45.77	27.90	27.67	3.1537	1797.6
***Quercus***	***Quercus prinus***	0.57	45.92	29.04	28.55	2.4434	1392.7
***Quercus***	***Quercus prinus***	0.57	49.25	29.33	29.26	4.3448	2476.5
***Quercus***	***Quercus prinus***	0.57	49.61	28.21	27.66	3.4979	1993.8
***Quercus***	***Quercus rubra***	0.56	12.95	17.00	16.91	0.1681	94.1
***Quercus***	***Quercus rubra***	0.56	14.60	17.50	17.16	0.2639	147.8
***Quercus***	***Quercus rubra***	0.56	22.82	19.80	19.42	0.5074	284.1
***Quercus***	***Quercus rubra***	0.56	26.42	21.84	21.66	0.7446	417.0
***Quercus***	***Quercus rubra***	0.56	33.70	30.86	30.73	1.6514	924.8
***Quercus***	***Quercus rubra***	0.56	38.72	30.01	29.51	2.2446	1257.0
***Quercus***	***Quercus rubra***	0.56	39.20	29.03	28.95	2.1682	1214.2
***Quercus***	***Quercus rubra***	0.56	46.68	28.68	28.09	3.7137	2079.7
***Quercus***	***Quercus rubra***	0.56	50.10	28.13	27.75	3.6146	2024.2
***Quercus***	***Quercus rubra***	0.56	52.93	28.68	28.27	3.2259	1806.5
***Quercus***	***Quercus rubra***	0.56	54.29	26.31	26.05	3.2712	1831.9
***Quercus***	***Quercus rubra***	0.56	60.00	31.05	30.62	5.2432	2936.2
***Quercus***	***Quercus rubra***	0.56	65.09	31.91	31.81	7.1455	4001.5
***Quercus***	***Quercus rubra***	0.56	68.56	31.17	31.16	4.8611	2722.2
***Quercus***	***Quercus rubra***	0.56	70.50	30.59	29.38	8.8624	4962.9
***Quercus***	***Quercus rubra***	0.56	91.20	30.67	30.58	12.7166	7121.3
***Quercus***	***Quercus rubra***	0.56	92.60	30.73	29.99	9.9249	5557.9

## 2 Experimental design, materials, and methods

We detail the terrestrial LiDAR sampling and post-processing method thoroughly in Refs. [1], [3]. A summary of the steps required to derive volume and biomass estimates from raw TLS data are outlined below.

Field-based measurements of DBH were collected during the same sampling season as the TLS acquisitions and used to validate the TLS-derived estimates. The presented tree measurements were collected across 14 1/10th ha circular plots. At each plot, we collected 5–6 TLS scans with a resolution totaling 28.2 pulses per scan, positioned approximately at the center and the four cardinal directions. Scans were registered using multiple registration points with an average error of less than 1 cm.

**Fig. 2:**
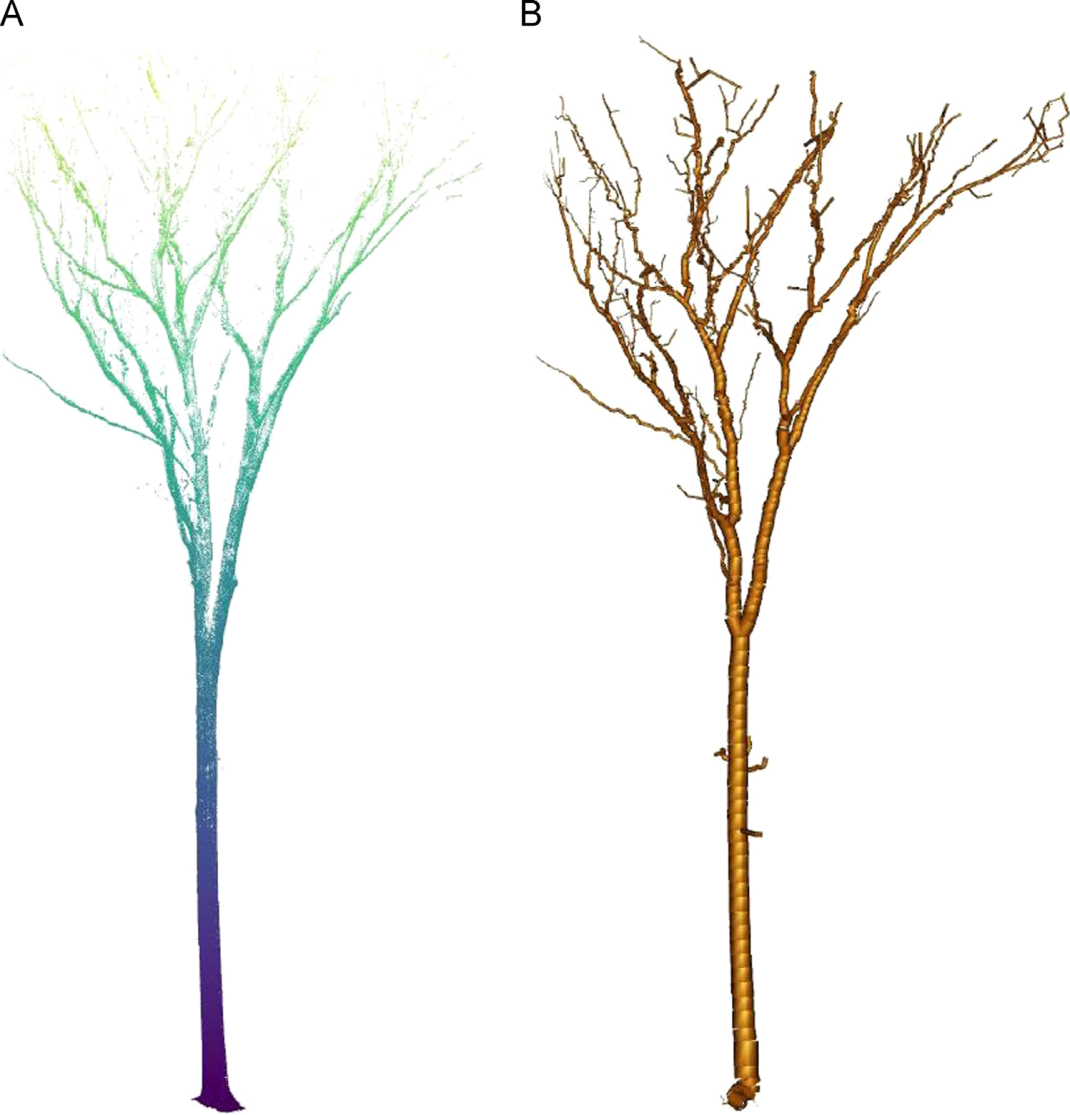
Example terrestrial LiDAR [A] point cloud and associated [B] three dimensional model. The modelling procedure allows for estimates of tree level diameter, height, volume, and biomass for use in allometric equation development.

Registered point cloud data were imported into Computree software [4] and SimpleTree [5] to estimate tree dimensions and create the volumetric tree models (Fig. 2). Tree diameter is estimated by fitting a cylinder at DBH height (1.3 m above ground). Tree height is defined as the distance from the lowest point to the highest point in the tree point cloud. Tree length is calculated similar to height, but, instead, measures the length of the approximate axis of the tree, from tree trunk to crown top. The volumetric tree models are derived from a series of three-dimensional cylinder objects fit to the LiDAR point cloud for estimates of trunk and branch diameter across the tree or interest. Total tree volume was derived by summing all cylinder fits on a per-tree basis. Average species specific wood density was used to convert estimates of wood volume into biomass. While average wood density is given in these data, regionally-derived species-specific wood density can easily be substituted for alternative biomass estimates.
